# Late gadolinium enhancement in areas with electrically fractionated potentials during sinus rhythm in patients with atrial fibrillation

**DOI:** 10.1007/s00380-025-02515-9

**Published:** 2025-02-08

**Authors:** Yuya Suzuki, Kunihiko Kiuchi, Mitsuru Takami, Kimitake Imamura, Jun Sakai, Toshihiro Nakamura, Atsusuke Yatomi, Yusuke Sonoda, Hiroyuki Takahara, Kazutaka Nakasone, Kyoko Yamamoto, Kenichi Tani, Hidehiro Iwai, Yusuke Nakanishi, Mitsuhiko Shoda, Shogo Yonehara, Atushi Murakami, Ken-ichi Hirata, Koji Fukuzawa

**Affiliations:** 1 Division of Cardiovascular Medicine, Department of Internal Medicine, Kobe University Graduate School of Medicine, 7-5-2 Kusunoki-cho, Chuo-ku, Kobe, Hyogo 650-0017 Japan; 2 Section of Arrhythmia, Division of Cardiovascular Medicine, Department of Internal Medicine, Kobe University Graduate School of Medicine, 7-5-2 Kusunoki-cho, Chuo-ku, Kobe, Hyogo 650-0017 Japan

**Keywords:** AEFP, Late gadolinium enhancement MRI, Atrial fibrillation, Left atrium, Atrial voltage

## Abstract

The areas with electrically fractionated potentials (AEFP) during sinus rhythm are related to non-pulmonary vein triggers and may serve as substrates of atrial fibrillation (AF) maintenance. However, the histological properties of these compounds remain unclear. Therefore, we aimed to evaluate the late gadolinium enhancement (LGE) properties of AEFP in patients with AF. We enrolled 15 patients with AF who had undergone LGE magnetic resonance imaging before catheter ablation. AEFP in the left atrium was detected using the HD-Grid and NavX systems after pulmonary vein isolation. We compared LGE properties between AEFP and the surrounding non-fractionated areas (non-AEFP). LGE heterogeneity and density were evaluated through entropy (LGE entropy) and the volume ratio of the enhancement voxel (LGE volume ratio), respectively. Thirty-three AEFP were detected in the left atrium. LGE entropy and LGE volume ratio were significantly higher in AEFP than in non-AEFP [LGE entropy: 6.2 (6.1–6.4) vs. 5.9 (5.8–6.0), *p* ≤ 0.0001; LGE volume ratio: 23.0% (17.2–29.0%) vs. 10.4% (3.4–20.2%), *p* ≤ 0.0001]. The atrial voltages did not differ [2.4 (1.3–3.7) vs. 2.5 (1.9–3.1) mV, *p* = 0.96]. AF recurrence was more significantly found in patients with more than three AEFP than in those without it (log-rank test: *p* = 0.009). AEFP is likely to be distributed in heterogeneous and moderate LGE areas, regardless of the atrial voltage.

## Introduction

Pulmonary vein isolation (PVI) is the standard ablation strategy for patients with atrial fibrillation (AF). Several adjunctive therapies for AF refractory to PVI have been reported [1, 2]. The procedure that should follow PVI remains unclear. However, non-pulmonary vein (PV) trigger ablation is the only Class IIa recommendation in the 2017 HRS/EHRA/ECAS/APHRS/SOLAECE expert statement [3].

Successful elimination of non‐PV triggers can improve the recurrence rate of AF ablation. However, the recurrence rate is higher if non-PV triggers remain in patients with AF [4, 5]. The main challenge in non-PV trigger ablation is the difficult mapping technique. Therefore, the novel mapping strategy using fractionated potentials in the atrium during sinus rhythm can help identify non-PV triggers [6]. Non-PV triggers are predominant at the site where the fractionated potential is observed in the sinus rhythm. Therefore, the new strategy utilizing areas with electrically fractionated potentials (AEFP) in the atrium during sinus rhythm facilitates the identification of non-PV triggers more than conventional mapping.

However, the histological properties of AEFP in the atrium during sinus rhythm remain unclear. Late gadolinium enhancement–magnetic resonance imaging (LGE–MRI) has reportedly been used to delineate fibrosis of the left atrium (LA) [7]. Therefore, we aimed to evaluate the LGE properties of AEFP in LA during sinus rhythm in patients with AF.

## Materials and methods

A total of 15 consecutive patients with paroxysmal (*n* = 11) and persistent (*n* = 4) AF who underwent a first-time catheter ablation and LGE–MRI before ablation were enrolled in this study. This retrospective study complied with the principles of the Declaration of Helsinki and was approved by the Ethics Committee of Kobe University Hospital (No. B220207). The patients consented to the use of their anonymized clinical data for research purposes through an opt-out process.

### MRI acquisition

Before AF ablation, all patients underwent contrast-enhanced MRI using a 1.5‐T MR system (Achieva; Philips Medical, Best, The Netherlands) equipped with a 5-channel cardiac coil. We have previously reported on this scan technique [8]. First, contrast-enhancement magnetic resonance angiography (CE‐MRA) of the PV-LA anatomy was acquired with a breath holding three‐dimensional (3D) fast field echo (FFE) sequence in the coronal plane during the first pass of a contrast agent (gadobutrol, Gadovist; Bayer Yakuhin, Osaka, Japan) injection at a dose of 0.1 mmol/kg [9]. Scanning in the coronal plane reduced the number of acquisition slices and shortened the breath holding time. Second, LGE–MRI of the LA with PVs was acquired using a 3D inversion recovery, respiration navigated, electrocardiogram-gated T1‐FFE sequence in the transverse plane 15 min after injecting the contrast [10]. The typical parameters were as follows: repetition time/echo time = 4.7/1.5 ms, voxel size = 1.43 × 1.43 × 2.40 mm (reconstructed to 0.63 × 0.63 × 1.20 mm), flip angle = 15°, SENSE = 1.8, and 80 reference lines. The inversion time was set to 280–320 ms using a Look‐Locker scan. For patients with AF, data were acquired with the shortest trigger delay for cardiac synchronization. However, for patients with sinus rhythm, data were acquired during the mid-diastolic phase of the left ventricle. The typical scan time for the LGE–MRI was 7–12 min, depending on the patient’s heart rate and respiratory pattern. Third, CE‐MRA and LGE–MRI scans were transferred to a customized software (MRI LADE Analysis; PixSpace Inc., Kitakyushu, Japan) for further image post‐processing and analysis.

### 3D visualization and assessment of tissue properties

We adopted the same protocol as that in our previous study for a more sensitive detection of weak LGE areas [11]. The 3D visualization method for LGE was as follows: first, the LA on LGE–MRI was segmented semi-manually by contouring the endocardial and epicardial borders of the atrium, including PVs. Second, the mean value and the standard deviation (SD) of the voxel intensity were measured on the “healthy” LA wall where no hyper-enhanced areas on LGE-MRA were observed. Third, a voxel intensity histogram analysis of the LA wall identified LGE as intensities ≤1 SD on the “healthy” LA wall. Furthermore, the degree of intensity was categorized using color-coded scaling (green: >1 SD; yellow: 2–3 SD; red: >3 SD). Finally, 3D reconstructions of the color-coded LGE and volume-rendered LA and PV images generated from CE-MRA were fused semi-automatically. Atrial fibrosis was defined as an LGE site with a signal intensity >1 SD.

To evaluate tissue properties, the fibrotic heterogeneity of the tissue was defined as the value of entropy (LGE entropy) using the Shannon formula:

$${\text{LGE}}{\text{-}}{\text{entropy}} = \sum\limits_{i} {Pi\,\ln (Pi)}$$

where *Pi* is the fraction of elements neighboring the *i*th element, a tissue type different from that of the *i*th element. The fibrotic density of the tissue was defined as the volume ratio of LGE signal intensity >1 SD (LGE volume ratio). LGE entropy and density were automatically calculated using a customized software (MRI LADE Analysis; PixSpace Inc., Kitakyushu, Japan).

### Ablation procedure

Before AF ablation, transesophageal echocardiography was performed to exclude thrombus formation. Patients were examined under moderate sedation while breathing spontaneously. Standard electrode catheters were placed at the right ventricular apex and coronary sinus, and a single transseptal puncture was performed. Unfractionated heparin was administered in bolus form before transseptal puncture to maintain an activated clotting time >350 s. Mapping and ablation were performed using an Eniste system (Abbott Laboratories, IL, USA) as a guide after integrating a 3D model of the LA and PV anatomy obtained from the pre-interventional MRI. Before ablation, a high-density mapping catheter (Advisor HD-Grid; Abbott Laboratories) and the ablation catheter–reconstructed LA posterior anatomy were aligned with the MRI. First, we performed PVI. After confirming the bidirectional block of PVs, a stimulation protocol (burst pacing from the coronary sinus at 300, 250, and 200 ms for 10 s each) was performed to test for inducibility. When AF was induced, the patient was cardioverted, and the procedure was terminated. No non-PV triggers were identified, and no further RF applications were performed on AEFP. Additional ablation of the cavotricuspid isthmus was performed only if a typical right atrial flutter was previously documented or induced by burst pacing at the end of the procedure.

### AEFP and low voltage

After PVI, we performed high-density voltage mapping of the LA using Advisor HD-Grid (Abbott Laboratories) during sinus rhythm. The low-voltage area (LVA) in the LA during sinus rhythm was defined as the bipolar voltage <0.5 mV.

We defined AEFP as follows: using the Fraction map of the Ensite system, we evaluated the atrial potential in the sinus rhythm. In the modified setting of the fraction map (Fractionation Threshold: 4, Sensitivity: 0.04 mV, Width: 3 ms, and Refractory: 6 ms), we delineated the areas where the number of fragmented potentials was >4 as AEFP. Further, to evaluate the distribution of AEFP, the entire LA region after PVI was divided into the following segments: roof, anterior, posterior, lateral, bottom, and septum.

### Relationship between LGE properties and atrial voltage on AEFP

We compared LGE properties between AEFP and control areas (non-AEFP). We semi-manually delineated one non-AEFP, LA without AEFP, and enhancement using LGE–MRI. Moreover, we compared atrial voltages between AEFP and non-AEFP.

### Relationship between AEFP and AF recurrence

After ablation, patients were monitored every 1–3 months. AF recurrence was defined as AF of a documented duration ≥30 s. We investigated the relationship between AEFP and AF recurrence.

Furthermore, to assess the ablation impact on the LGE area associated with AEFP, we compared the rhythm outcome between the current 15 cases with PVI alone and case-matched 15 cases with PVI plus additional ablation on the LGE areas associated with AEFP.

### Statistical analysis

Data are presented as means and SDs or proportions. Variables were compared using the chi-square or Fisher’s exact test, as appropriate. Receiver operating characteristic (ROC) curves were used to determine the LGE entropy and LGE volume ratio, which provided the best sensitivity and specificity for AEFP. A Kaplan–Meier analysis was performed to assess recurrence-free survival, and the log-rank test was used to compare the groups. To compare the rhythm outcome between the PVI plus additional ablation on LGE areas and PVI alone, sex-, left atrial diameter- (LAD), and left ventricular ejection fraction- (LVEF)—matched group was selected from the Kobe University AF registry as additional ablation group. All analyses were performed using IBM^®^SPSS^®^ software, version 26 (IBM Corporation, Armonk, NY, USA), and statistical significance was set at *p* < 0.05.

## Results

### Patients and procedural characteristics

Table 1 shows patient and procedural characteristics. Mean age, left atrial dimension, and left ventricular ejection fraction were 64.5 ± 7.9 years, 37.5 ± 4.1 mm, and 60.8 ± 8.0%, respectively. Eleven (73.3%) of the 15 patients had paroxysmal AF, and all patients underwent initial AF catheter ablation. The mean time from MRI acquisition to AF ablation was 141.2 ± 96.9 days.

**Table 1:** Baseline characteristics

	Total (*n* = 15)
Age (years old)	64.5 ± 7.9
Male, *n* (%)	13 (86.7)
BMI (kg/m^2^)	23.6 ± 2.5
Cre (mg/dL)	0.84 ± 0.16
BNP (pg/mL)	67.8 ± 61.8
CHADS_2_ score	1.07 ± 0.96
Paroxysmal AF, *n* (%)	11 (73.3)
Persistent AF, *n* (%)	4 (26.7)
Initial AF ablation (%)	15 (100)
LAD (mm)	37.5 ± 4.1
LVEF (%)	60.8 ± 8.0
LAA flow (m/s)	56 ± 24.5
The time from MRI acquisition to the ablation (days)	141.2 ± 96.9

*BMI* body mass index, *Cre* creatinine, *BNP* brain natriuretic peptide, *AF* atrial fibrillation, *LAD* left atrial diameter, *LVEF* left ventricular ejection fraction, *LAA* left atrial appendage, *MRI* magnetic resonance imaging

### LGE properties on AEFP and non-AEFP

**Fig. 1:**
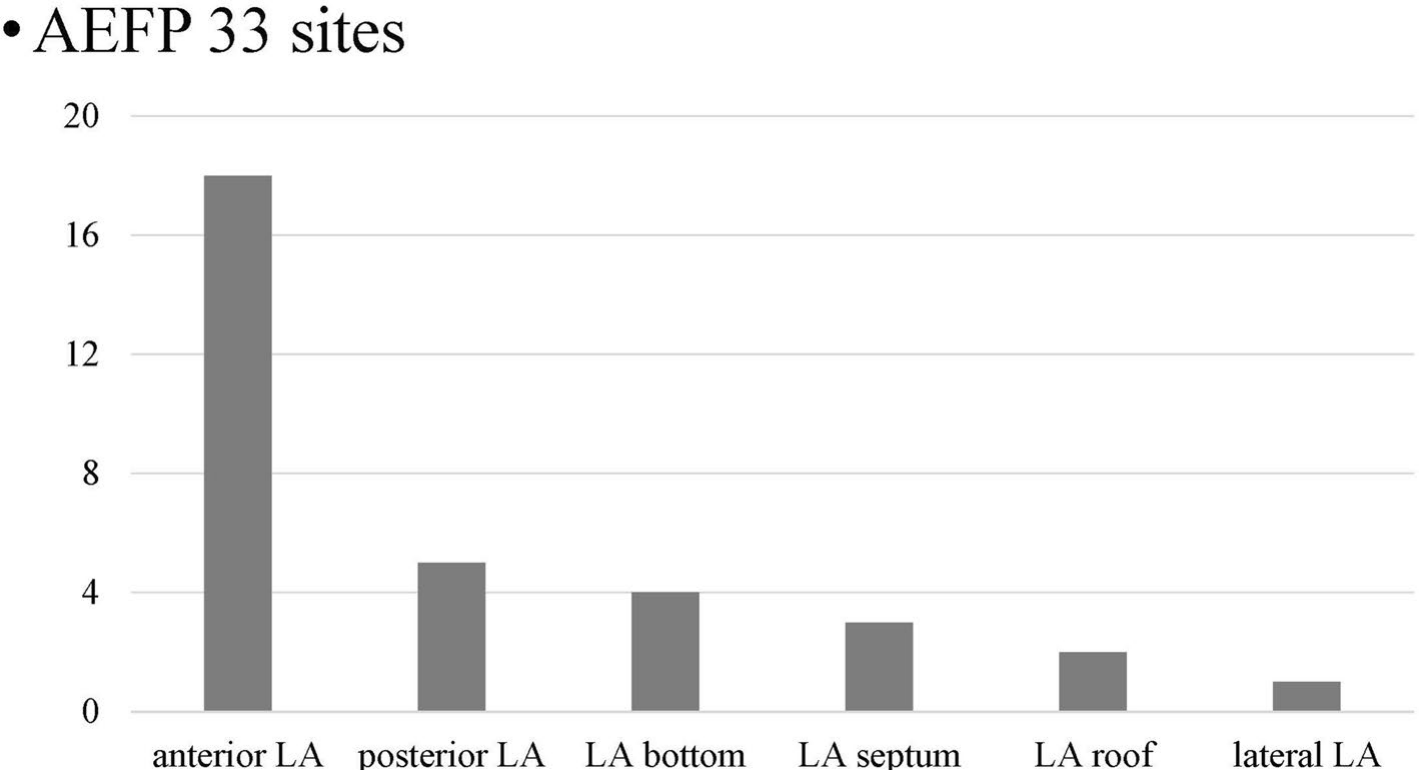
Distribution of AEFP. AEFP, area with electrically fractionated potential; LA, left atrium

All patients had at least one AEFP and LGE-positive area. The number of the AEFP in each patient ranged from 1 to 5. Overall, 33 AEFP were found in the LA after PVI, and 62 non-AEFP were delineated. Figure 1 shows the distribution of AEFP. AEFP of the LA post-PVI was mostly observed in the anterior LA (54.5%). Table 2 shows the LGE properties of LGE entropy and LGE volume ratio between AEFP and non-AEFP. The LGE entropy was significantly higher in AEFP than in non-AEFP (LGE entropy: 6.2 [6.1–6.4] vs. 5.9 [5.8–6.0], *p* < 0.0001). LGE volume ratio was also significantly higher in AEFP than in non-AEFP (LGE volume ratio: 23.0% [17.2–29.0%] vs. 10.4% [3.4–20.2%], *p* ≤ 0.0001). A ROC curve analysis yielded an optimal cutoff value of 6.0 and 11.5% for LGE entropy and LGE volume ratio, respectively. Regarding the optimal LGE entropy, sensitivity, specificity, and positive and negative predictive values for the cutoff values were 100%, 79.0%, 71.7%, and 100%, respectively. However, for the optimal LGE volume ratio, sensitivity, specificity, and positive and negative predictive values for the cutoff values were 87.9%, 67.7%, 59.2%, and 91.3%, respectively.

**Table 2:** LGE properties and atrial voltages between AEFP and non-AEFP

	AEFP (*n* = 33)	Non-AEFP (*n* = 62)	*p* value
*LGE–MRI*			
LGE entropy	6.2 (6.1–6.4)	5.9 (5.8–6.0)	<0.0001
LGE volume ratio (%)	23.0 (17.2–29.0)	10.4 (3.4–20.2)	<0.0001
*Atrial voltage*			
Mean voltage (mL)	2.4 (1.3–3.7)	2.5 (1.9–3.1)	0.96
LVA (<0.5 mV) (%)	5 (15.2)	6 (9.7)	0.51

*AEEP* area with electrically fractionated potential, *LGE* late gadolinium enhancement, *LVA* low-voltage area

**Fig. 2:**
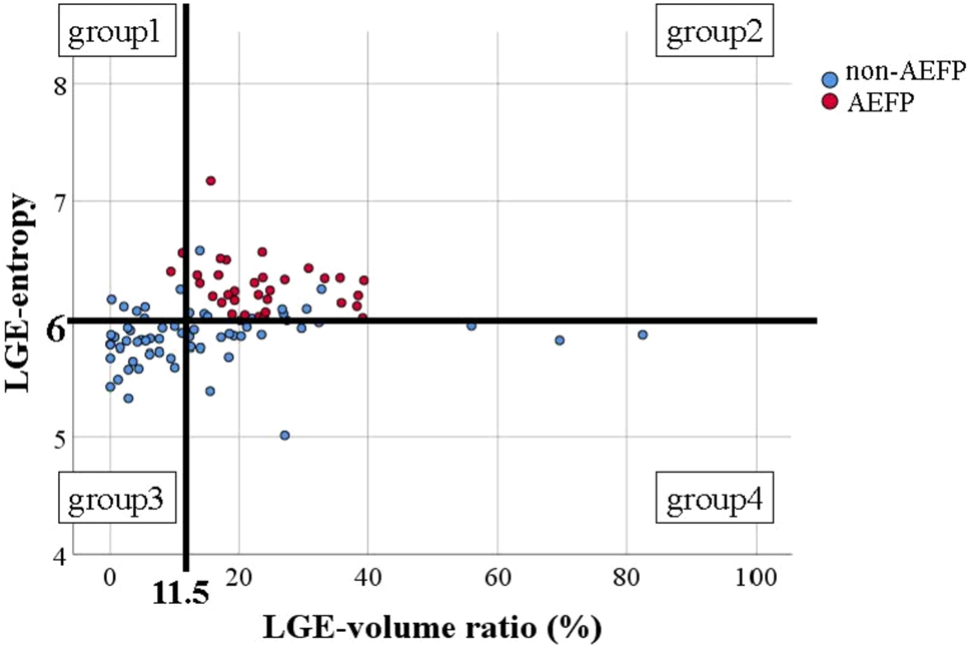
Distribution of AEFP and non-fractionated areas according to LGE entropy and LGE volume ratio. AEFP (red) and non-AEFP (blue). AEFP and non-AEFP are classified into four groups (groups 1–4) according to the ratio of LGE entropy of 6.0 and LGE volume of 11.5%. AEFP, area with electrically fractionated potential; LGE, late gadolinium enhancement

**Fig. 3:**
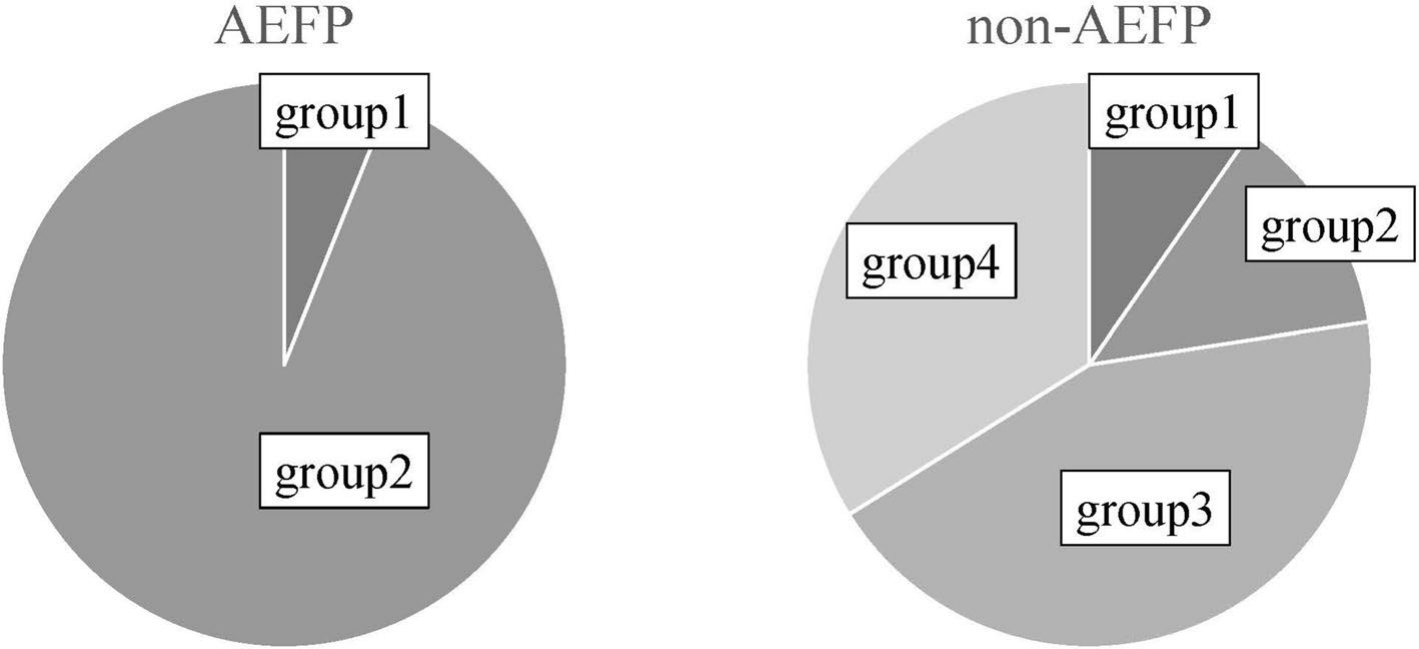
Proportion of fibrotic tissue properties between AEFP and non-AEFP. AEFP, area with electrically fractionated potential

Based on an LGE entropy of 6.0 and LGE volume ratio of 11.5%, LGE properties were classified into four groups: heterogenous healthy area (Group 1 [G1]: LGE entropy ≥6.0 and LGE volume ratio <11.5%), heterogenous LGE area (Group 2 [G2]: LGE entropy ≥6.0 and LGE volume ratio ≥10%), homogenous healthy area (Group 3 [G3]: LGE entropy <6.0 and LGE volume ratio <11.5%), and homogenous LGE area (Group 4 [G4]: LGE entropy <6.0 and LGE volume ratio ≥11.5%). Figure 2 shows the distribution of AEFP and non-AEFP according to the LGE entropy and LGE volume ratio. Notably, AEFP was not observed in regions with an LGE volume ratio ≥40%. Figure 3 shows the proportions of AEFP in each group.

As shown in Fig. 3, AEFP predominantly consisted of G2 and, subsequently, G1. However, non-AEFP predominantly consisted of G3 and subsequently, G4 (AEFP: 2 [6.1%] in G1, 31 [93.9%] in G2, 0 [0.0%] in G3, 0 [0%] in G4; non-AEFP: 6 [9.7%] in G1, 8 [12.9%] in G2, 27 [43.5%] in G3, 21 [33.9%] in G4; *p* ≤ 0.0001).

### Relationship between the atrial voltage and AEFP

**Fig. 4:**
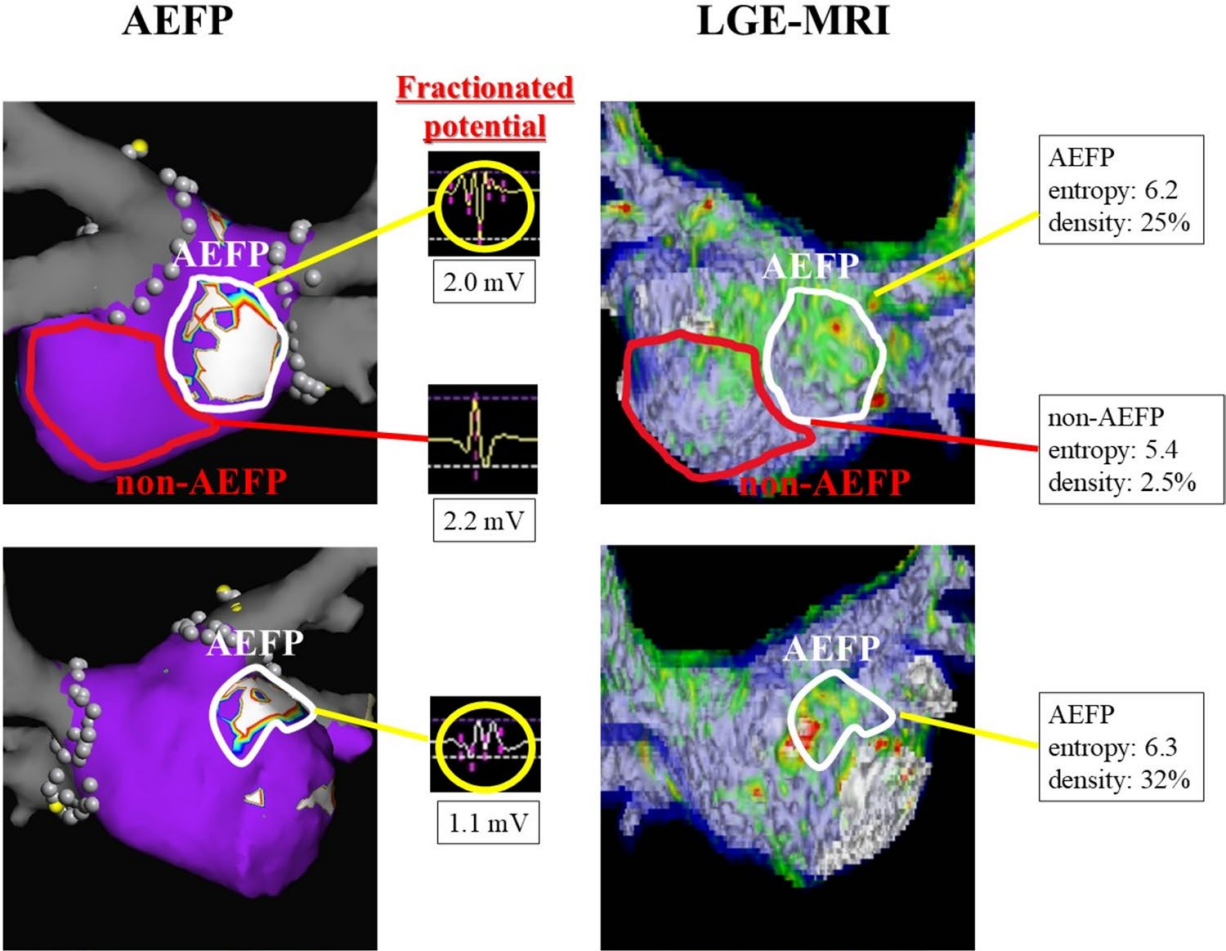
Representative case. The left-hand side of the figure shows AEFP. Areas with a fractionation threshold ≥4 are shown in white. The right-hand side shows LGE properties. AEFP, area with electrically fractionated potential; LGE, late gadolinium enhancement

Table 2 shows the proportion of LVA in AEFP and non-AEFP. The atrial voltages were comparable between AEFP and non-AEFP (2.4 [1.3–3.7] vs. 2.5 [1.9–3.1] mV, *p* = 0.96). The LVA was also comparable between AEFP and non-AEFP (5 [15.2%] vs. 6 [9.7%], *p* = 0.5). Figure 4 shows a representative example.

### Relationship between AF recurrence and AEFP

**Fig. 5:**
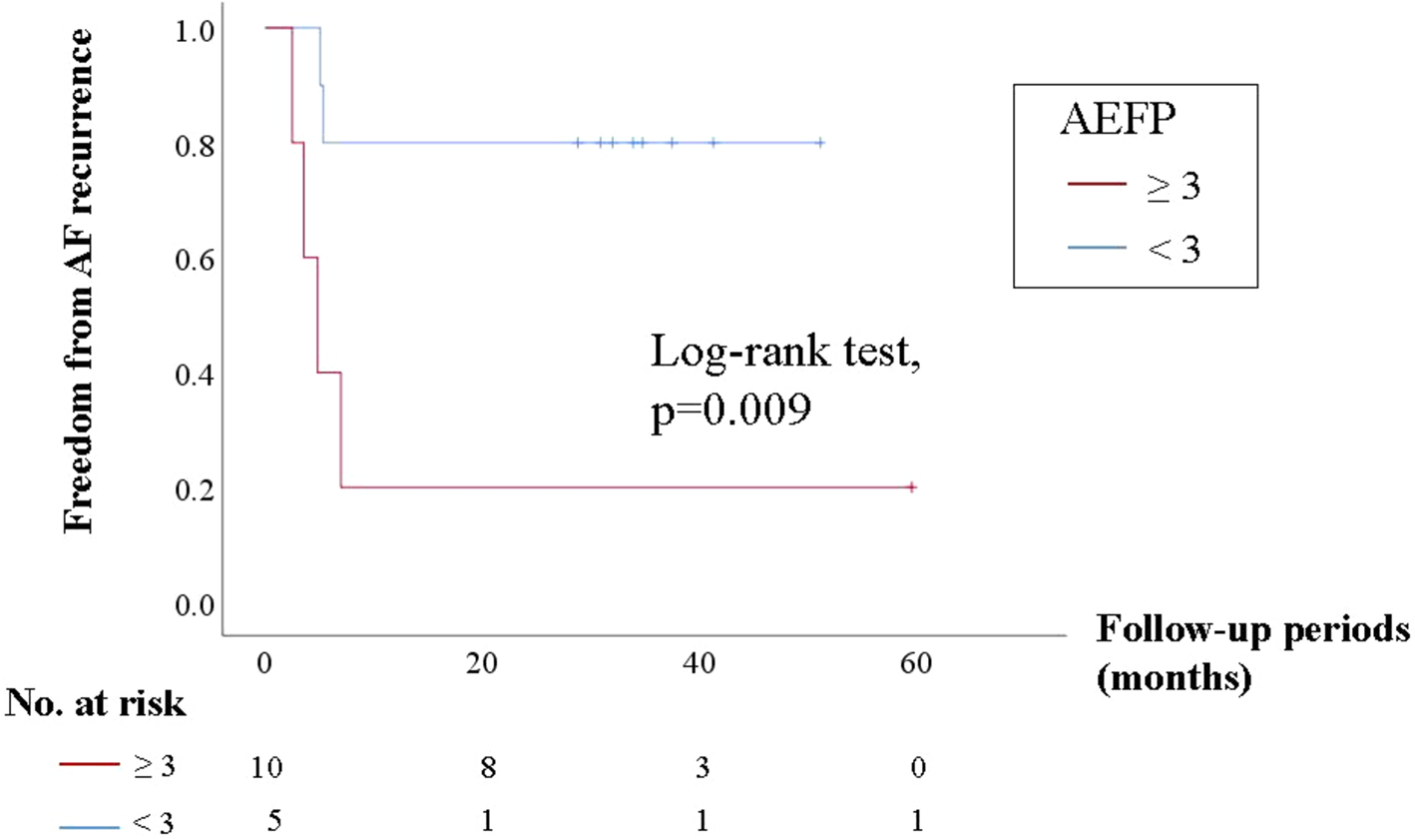
Kaplan–Meier analysis of time to first atrial ablation recurrence after catheter ablation

AF recurrence was observed in 6 (40%) of 15 patients in the mean follow-up period of 36 months. The type of recurrent AF was PAF in 4 and non-PAF in 2 patients, respectively. Patients with more than three AEEP had more AF recurrence events than those with less than three AEEP (4 of 5 patients [80%] vs. 2 of 10 patients [20%], *p* = 0.09). Kaplan–Meier analysis showed a significant difference in cumulative hazard curves for AF recurrence between patients with more than three AEFP and those with less than three AEFP (log-rank test: *p* = 0.009; Fig. 5). Of interest, in the case-matched group of additional ablations on the LGE areas, non-PV triggers were found in 6 (40%) of 15 patients. Nevertheless, the rhythm outcome after PVI plus additional ablation on LGE areas was significantly better than PVI alone (AF rec: 6(40%) of 15 pts vs. 1 (7%) of 15 pts, log-rank *p* = 0.038).

## Discussion

### Main findings

The LGE entropy and LGE volume ratio was significantly higher in AEFP than in non-AEFP. However, the atrial voltages did not differ between AEFP and non-AEFP.

### Fibrotic remodeling and non-PV triggers

The relationship between pathophysiological properties and non-PV triggers remains unclear.

In previous studies, myofibroblasts depolarized cardiomyocytes via heterocellular electronic interactions through gap junctions and generated ectopic activity in the myocardium with fibrotic remodeling [12, 13]. In this study, AEFP was distributed on high LGE entropy and mild LGE volume ratio areas, which indicates that heterogeneous fibrosis might be critical in non-PV triggers. Fibrosis is prevalent in patients with AF [14]. Vulnerability to arrhythmia increases with the amount of fibrosis, and the texture of fibrosis is also crucial [15]. Patchy fibrosis mainly impairs transverse conduction. However, it leaves longitudinal conduction largely unaffected, resulting in zig-zag activation patterns. Diffuse fibrosis does not significantly influence the activation pattern; however, it causes an overall slowing of conduction. In an adult rabbit, the presence of patchy fibrosis generates fragmented potential [16]. However, the presence of high-frequency fractionated near-field components next to lower-frequency healthy voltage is relatively common in patients with AF [17].

### Discrepancy of the LGE property and atrial voltage

Previous studies have shown a relationship between the LA bipolar endocardial voltage and LGE enhancement. Low-voltage areas are associated with the extent of LGE enhancement [18, 19]. However, a discrepancy between voltage mapping and LGE enhancement has been reported.

This discrepancy was explained by the possibility that the peak-to-peak bipolar voltage criterion causes an abnormal electrocardiogram to be misinterpreted as a normal electrocardiogram [20]. An association between complex continuous fractionated atrial electrogram, a fragmented potential during AF, and LGE enhancement has been reported previously [19]. Recently, Kuo et al. reported the relationship between electrogram (EGM) fractionation during sinus rhythm and LGE enhancement [17]. These EGM fractionations specifically correlated with de novo LGE but did not correlate with previous ablation lesions. They suggested that sites with EGM fractionation during sinus rhythm exhibited LGE enhancement despite normal voltage, which might signify structural changes, such as expanded extracellular space, fat infiltration, or inflammation. They also suggested that these atrial substrates enhance functional re-entry and AF perpetuation. In this previous study, among all fractionated EGMs during sinus rhythm, approximately 30% were located in low-voltage areas and 70% in normal voltage areas. In our study, approximately 15% of AEFP was located in low-voltage areas, and AEFP was not associated with atrial voltage. Therefore, the fragmented potential during the sinus rhythm may reflect the change in the substrate that does not appear as a change in voltage. A previous study reported a relationship between the signal intensity of LGE–MRI and fractionated EGMs during sinus rhythm. We also recently reported the relationship between heterogeneity and the burden of LGE enhancement and fragmented potential during sinus rhythm using LGE entropy.

### Importance of AEFP as an AF substrate

In a previous study, ablation of AEFP for patients with AF recurrence after AF ablation decreased the recurrence rate of atrial tachyarrhythmia [21]. We previously reported that fragmented LGE area (heterogenous, LGE volume ratio 10–50%) ablation for patients with AF could terminate AF or convert it to atrial tachycardia, and the rhythm outcome of this procedure was improved compared with that of a PVI [11]. We considered that the mechanism of the improved outcomes was ablation of heterogenous LGE areas as an intervention of the AF rotor. Using LGE–MRI and novel phase mapping system (ExTRa Mapping™) identifying AF rotor, we found that meandering re-entrant AF drivers could be due to heterogeneous fibrosis. This heterogeneous LGE site tended to have higher LGE entropy (>5.7) and was distributed in a mild LGE area (LGE volume ratio 10–50%) [22]. The previous computed model also showed that AF re-entrant drivers (rotors) were associated with high density and entropy of fibrosis on LGE–MRI [23]. In this study, AEFP was distributed in high LGE entropy (>6.0) and mild LGE volume ratio areas, which might indicate heterogeneous fibrosis. Moreover, no AEFP could be found in regions with an LGE‐volume ratio >40%. AEFP is likely to be distributed in heterogeneous and mild LGE areas. Moreover, in this study, AF recurrence events were highly observed in patients with more than three AEFP. Therefore, AEFP may be associated with non-PV triggers and the AF substrate.

### Clinical implications

Ablation of AEFP of the atrium during sinus rhythm is a novel and effective strategy for recurrent AF; however, mapping of AEFP has the following limitations: (1) only available in cases that can maintain sinus rhythm, (2) time-consuming, and (3) dependent on the operator’s skill in LA mapping. However, preoperative LGE–MRI can overcome these limitations by identifying Group 2 sites (high-entropy and mild LGE sites).

### Limitations

First, the sample size is relatively small. Second, the LGE site may have been overestimated on the posterior wall adjacent to the vertebrae and the anterior wall adjacent to the aortic cusp because of wall compression by those organs. Third, novel mapping of AEFP has some unknown limitations.

## Conclusion

AEFP are likely distributed in heterogeneous and moderate LGE areas, regardless of the atrial voltage. Therefore, AEFP might signify slight local structural remodeling.

## Data Availability

The deidentified participant data will not be shared.
